# Non-linear associations of the triglyceride-glucose index with thyroid function in a large Chinese population: a cross-sectional study

**DOI:** 10.3389/fnut.2026.1841938

**Published:** 2026-07-23

**Authors:** Minghui Sun, Xiaoyu Zhang, Xiao Guan

**Affiliations:** Health Management Center, Qilu Hospital, Cheeloo College of Medicine, Shandong University, Jinan, Shandong, China

**Keywords:** insulin resistance, non-linear association, threshold effect, thyroid function, triglyceride-glucose index

## Abstract

**Background:**

Previous studies have identified the triglyceride-glucose (TyG) index as a practical surrogate marker of insulin resistance and have suggested its association with several metabolic disorders. However, the association between the TyG index and thyroid function parameters has not yet been fully clarified. The present study was conducted to examine this association in Chinese adults from the general population.

**Methods:**

This cross-sectional study included 5,189 adults who underwent health examinations. Multivariable linear regression was performed to investigate the associations between the TyG index and thyroid function parameters, including free triiodothyronine (FT3), free thyroxine (FT4), and thyroid-stimulating hormone (TSH). Potential non-linear dose–response associations were explored by using restricted cubic spline (RCS) analyses, while threshold effects were evaluated using piecewise linear regression. Subgroup analyses were conducted to examine whether the associations varied across subgroups, and sensitivity analyses were performed in strictly euthyroid participants.

**Results:**

After full adjustment for potential confounders, the TyG index showed no clear linear association with FT3, whereas it was inversely associated with FT4. Non-linearity analyses indicated a significant inverted U-shaped association of the TyG index with FT3 (P for non-linearity < 0.001), the TyG index was positively associated with FT3 below the inflection point of 8.311 and negatively associated with FT3 above this point. In contrast, piecewise linear regression suggested a threshold effect for FT4 (P for likelihood ratio test = 0.016), with the TyG index showing a positive association below 7.591 and a negative association above this point. Additionally, the TyG index was positively associated with lnTSH after full adjustment. Subgroup analyses suggested potential effect modification for FT3 by sex, FT4 by thyroid status, and TSH by age. The non-linear patterns for FT3 and FT4 remained generally similar in sensitivity analyses restricted to strictly euthyroid participants.

**Conclusion:**

This study observed complex linear and non-linear associations between the TyG index and thyroid function parameters. These findings suggest a potential metabolic-thyroid association that warrants further evaluation in longitudinal studies.

## 1 Introduction

Insulin resistance, characterized by impaired cellular responsiveness to insulin, is closely involved in metabolic dysregulation (1). Accurate assessment of insulin resistance is important for evaluating metabolic risk and guiding disease management. Although the hyperinsulinemic–euglycemic clamp is widely regarded as the reference method for assessing insulin resistance (2), its use in routine clinical settings is limited by its invasive nature, high cost, and procedural complexity. In this context, the triglyceride-glucose (TyG) index (3–5), calculated from fasting blood glucose (FBG) and triglyceride (TG) concentrations, has been increasingly used as a practical surrogate marker of insulin resistance. Previous studies have suggested that the TyG index may perform as well as or better than traditional indices such as the homeostasis model assessment of insulin resistance, (HOMA-IR) in assessing metabolic risk across diverse populations (6–10).

Thyroid hormones are important regulators of systemic metabolic homeostasis and are involved in carbohydrate metabolism, lipid mobilization, and mitochondrial bioenergetics (11). An increasing body of evidence has shown associations between thyroid dysfunction and insulin resistance and other metabolic abnormalities. Both overt and subclinical hypothyroidism, characterized by elevated thyroid-stimulating hormone (TSH) levels with reduced or normal free thyroxine (FT4) levels, have been associated with metabolic syndrome (12), diabetes mellitus (13), and greater insulin resistance severity as assessed by HOMA-IR (14). In contrast, hyperthyroid states have also been associated with altered glucose homeostasis, potentially involving increased hepatic glucose production and altered glucose metabolism in peripheral tissues (15). Clinical evidence suggests complex associations between thyroid function and insulin resistance (16, 17), although existing findings remain inconsistent.

In previous studies of thyroid function, insulin resistance has commonly been estimated using HOMA-IR. However, relatively few studies have used the TyG index, and most of these studies have focused on its linear associations with thyroid function parameters, with limited exploration of potential non-linear or threshold effects. Therefore, the present study assessed the associations between the TyG index and thyroid function parameters in Chinese adults, with particular attention to potential non-linear patterns and threshold effects.

## 2 Methods

### 2.1 Study subjects

Participants aged 18 to 80 years who underwent routine health examinations from January to December 2023 were included in this cross-sectional study at the Health Management Center of Qilu Hospital, Shandong University. Participants were excluded if they met any of the following criteria: pregnancy or lactation; a history of thyroid surgery; current use of medications affecting thyroid function, glucose metabolism, or lipid profiles; severe hepatic or renal insufficiency; malignant tumors; known systemic autoimmune diseases or acute infectious diseases; or incomplete demographic or laboratory data. A total of 5,189 participants were included in the final analysis. The study was conducted in accordance with the Declaration of Helsinki and was approved by the Ethics Committee of Qilu Hospital, Shandong University.

### 2.2 Clinical and laboratory measurements

All participant data were extracted from the electronic medical record system of the Health Management Center. These data included demographic characteristics (age, sex, educational level), physical assessment data such as height, weight, body mass index (BMI), and blood pressure, laboratory parameters, and medical history. Anthropometric parameters were recorded by trained nurses following standardized protocols: height and weight were used to calculate BMI (kg/m^2^), and blood pressure was measured after at least 5 min of seated rest. Regarding lifestyle history, smoking was defined as having smoked at least 100 cigarettes during one’s lifetime, whereas alcohol consumption referred to an intake of at least 30 g per week for no less than 1 year.

After an overnight fast of no less than 8 h, venous blood samples were collected the next morning and analyzed in the hospital laboratory. FBG, liver function parameters including alanine aminotransferase (ALT), aspartate aminotransferase (AST), and gamma-glutamyl transferase (GGT); renal function markers including creatinine (Cr), blood urea nitrogen (BUN), and uric acid (UA); and lipids including TG, total cholesterol (TC), low-density lipoprotein cholesterol (LDL-C), and high-density lipoprotein cholesterol (HDL-C) were measured using a fully automated biochemical analyzer (Cobas c702, Roche Diagnostics). Serum concentrations of free triiodothyronine (FT3), FT4, TSH, thyroglobulin antibody (TgAb), and thyroid peroxidase antibody (TPOAb) were measured by electrochemiluminescence immunoassay using a Cobas e 801 instrument. The laboratory reference ranges were 0.270–4.200 μIU/mL for TSH and 11.90–21.60 pmol/L for FT4. Antibody positivity was defined as TPOAb ≥34 IU/mL or TgAb ≥115 IU/mL. The FT3/FT4 ratio was calculated as FT3 divided by FT4. Based on these parameters, participants were classified into four groups: euthyroid antibody-negative, defined as normal TSH and FT4 levels with negative antibodies; euthyroid antibody-positive, defined as normal TSH and FT4 levels with positive TPOAb and/or TgAb; hypothyroidism, including overt hypothyroidism (TSH > 4.200 μIU/mL and FT4 < 11.90 pmol/L) and subclinical hypothyroidism (TSH > 4.200 μIU/mL and normal FT4); and hyperthyroidism, including overt hyperthyroidism (TSH < 0.270 μIU/mL and FT4 > 21.60 pmol/L) and subclinical hyperthyroidism (TSH < 0.270 μIU/mL and normal FT4). The TyG index was calculated using the following formula: ln [fasting triglycerides (mg/dL) × fasting blood glucose (mg/dL)/2].

### 2.3 Statistical analysis

All statistical analyses were conducted using R (version 4.2.2). Variables with a normal distribution were presented as mean ± standard deviation. To assess intergroup differences across the TyG quartile groups, one-way analysis of variance was used. Skewed variables were presented as median (interquartile range [IQR]), and differences across groups were evaluated using the Kruskal–Wallis H test. Categorical variables were summarized as frequencies (percentages) and compared using the chi-square test. Given the skewed distribution of TSH, it was natural log-transformed (lnTSH) to approximate a normal distribution for further analysis.

The associations between the TyG index and thyroid function parameters were evaluated using multivariable linear regression models. Three models were constructed: no variables were adjusted for in Model 1; Model 2 adjusted for age and sex, while Model 3 adjusted for BMI, smoking, drinking, education, ALT, AST, GGT, UA, Cr, BUN, hemoglobin (Hb), TgAb, and TPOAb.

The rms package was used to fit restricted cubic spline (RCS) models to investigate potential dose–response associations between the TyG index and thyroid function parameters. Knots were placed at the 5th, 35th, 65th, and 95th percentiles of the TyG index, with the median as the reference. Overall effects and non-linearity were evaluated using the Wald chi-square test. To assess potential threshold effects, piecewise linear regression models were further fitted, and the model with the better fit was selected based on the likelihood ratio test (LRT). Subgroup analyses were conducted by age, sex, BMI, and thyroid function status to examine whether the associations differed across strata; interactions were assessed using LRT. Sensitivity analyses were conducted among strictly euthyroid, antibody-negative participants to assess the consistency of the results. In participants with available HbA1c data, additional sensitivity analyses were performed by further adjusting for HbA1c. Findings with a two-sided *p* value <0.05 were considered statistically significant.

## 3 Results

### 3.1 Characteristics of the study population

Baseline characteristics of the 5,189 participants, stratified by TyG index quartiles, are presented in Table 1 and Supplementary Table S1. Individuals in the highest quartile had a less favorable metabolic profile than those in the lowest quartile. Specifically, the Q4 group included older participants, a higher proportion of male participants, higher BMI and blood pressure, and higher proportions of smokers and alcohol consumers (all *p* < 0.001). Metabolic and biochemical profiles differed significantly across TyG quartiles. As the TyG index increased, FBG, TC, TG, LDL-C, ALT, AST, GGT, Cr, BUN, and UA were higher, whereas HDL-C decreased (all *p* < 0.001). Regarding thyroid function, TSH (*p* = 0.004), FT3 (*p* < 0.001), and the FT3/FT4 ratio (*p* < 0.001) increased across quartiles, whereas FT4 levels tended to decrease but did not reach statistical significance (*p* = 0.058). The distribution of thyroid status differed significantly (*p* = 0.001). Hypothyroidism was most frequent in the Q4 group, where its prevalence reached 7.5%.

**Table 1:** Baseline characteristics of the study participants stratified by TyG index quartiles.

Characteristics	TyG index				Overall (*N* = 5,189)	*p*-value
	Q1 (*N* = 1,298)	Q2 (*N* = 1,297)	Q3 (*N* = 1,297)	Q4 (*N* = 1,297)		
Age (years)	42.4 ± 12.5	46.6 ± 13.2	49.4 ± 12.9	50.4 ± 12.8	47.2 ± 13.2	<0.001
Sex						<0.001
Male	457 (35.2%)	665 (51.3%)	847 (65.3%)	1,005 (77.5%)	2,974 (57.3%)	
Female	841 (64.8%)	632 (48.7%)	450 (34.7%)	292 (22.5%)	2,215 (42.7%)	
BMI (kg/m^2^)	22.4 ± 3.0	24.4 ± 3.3	25.6 ± 3.3	26.8 ± 3.1	24.8 ± 3.6	<0.001
SBP (mmHg)	117.8 ± 16.1	125.1 ± 18.2	130.5 ± 18.3	135.2 ± 18.6	127.1 ± 19.0	<0.001
DBP (mmHg)	73.0 ± 10.5	77.3 ± 11.5	80.9 ± 11.8	84.4 ± 11.8	78.9 ± 12.2	<0.001
FT3 (pmol/L)	4.9 ± 0.9	5.1 ± 1.2	5.2 ± 1.6	5.3 ± 0.9	5.2 ± 1.2	<0.001
FT4 (pmol/L)	16.5 ± 3.0	16.4 ± 3.0	16.6 ± 3.9	16.2 ± 2.3	16.4 ± 3.1	0.058
FT3/FT4	0.30 ± 0.05	0.32 ± 0.05	0.32 ± 0.05	0.33 ± 0.06	0.32 ± 0.05	<0.001
TSH (μIU/mL)	1.86 (1.31, 2.54)	1.83 (1.32, 2.62)	1.90 (1.38, 2.63)	1.93 (1.41, 2.72)	1.88 (1.35, 2.62)	0.004
TPOAb (IU/mL)	11.41 (8.88, 15.49)	11.53 (9.04, 15.44)	11.65 (8.99, 15.35)	11.68 (9.05, 15.65)	11.56 (8.99, 15.48)	0.776
TgAb (IU/mL)	16.57 (14.82, 19.73)	16.30 (14.49, 18.87)	16.20 (14.59, 18.81)	15.98 (14.38, 18.19)	16.26 (14.57, 18.81)	<0.001
Thyroid Status						0.001
Euthyroid	1,020 (78.6%)	1,031 (79.5%)	1,032 (79.6%)	1,058 (81.6%)	4,141 (79.8%)	
Euthyroid with antibody-positivity	160 (12.3%)	136 (10.5%)	129 (9.9%)	92 (7.1%)	517 (10.0%)	
Hyperthyroidism	28 (2.2%)	16 (1.2%)	19 (1.5%)	17 (1.3%)	80 (1.5%)	
Hypothyroidism	72 (5.5%)	78 (6.0%)	88 (6.8%)	97 (7.5%)	335 (6.5%)	

Continuous variables are presented as mean ± SD or median (interquartile range), and categorical variables are presented as number (percentage). Euthyroid with antibody-positivity was defined as having normal thyroid function but with positivity for TPOAb and/or TgAb. BMI, body mass index; SBP, systolic blood pressure; DBP, diastolic blood pressure; FT3, free triiodothyronine; FT4, free thyroxine; TSH, thyroid-stimulating hormone; TPOAb, thyroid peroxidase antibody; TgAb, thyroglobulin antibody; TyG index, triglyceride-glucose index.

### 3.2 Association between the TyG index and thyroid function parameters

The linear regression results for the associations between the TyG index and FT3, FT4, the FT3/FT4 ratio, and lnTSH are presented in Table 2. For FT3 and FT4, different association patterns were observed. For FT3, although the TyG index was positively associated with FT3 in the crude model, this association was attenuated after multivariable adjustment and was no longer statistically significant in the fully adjusted model (*β* = −0.025, 95% CI: −0.085, 0.034, *p* = 0.404). When the TyG index was analyzed by quartiles, no significant trend across quartiles was observed for FT3 after full adjustment (P for trend = 0.902). In contrast, after full adjustment for potential confounders (Model 3), the TyG index was significantly and negatively associated with FT4 levels (*β* = −0.264, 95% CI: −0.426, −0.103, *p* = 0.001). Compared with participants in the lowest TyG quartile, those in the highest quartile had lower FT4 levels (*β* = −0.602, 95% CI: −0.893, −0.310, *p* < 0.001), with a significant trend across quartiles (P for trend < 0.001).

**Table 2:** Association between TyG index and thyroid function parameters.

Outcome	TyG index	Model 1 *β* (95% CI)	*P*-value	Model 2 *β* (95% CI)	*P*-value	Model 3 *β* (95% CI)	*P*-value
FT3	Continuous	0.191 (0.141, 0.242)	<0.001	0.091 (0.039, 0.143)	<0.001	−0.025 (−0.085, 0.034)	0.404
	Q4 vs. Q1	0.351 (0.258, 0.444)	<0.001	0.190 (0.094, 0.285)	<0.001	−0.005 (−0.113, 0.103)	0.927
	P for trend		<0.001		<0.001		0.902
FT4	Continuous	−0.068 (−0.199, 0.063)	0.310	−0.232 (−0.370, −0.093)	0.001	−0.264 (−0.426, −0.103)	0.001
	Q4 vs. Q1	−0.257 (−0.498, −0.015)	0.037	−0.534 (−0.789, −0.279)	<0.001	−0.602 (−0.893, −0.310)	<0.001
	P for trend		0.139		<0.001		<0.001
FT3/FT4	Continuous	0.014 (0.011, 0.016)	<0.001	0.011 (0.008, 0.013)	<0.001	0.004 (0.001, 0.007)	0.005
	Q4 vs. Q1	0.028 (0.023, 0.032)	<0.001	0.023 (0.018, 0.027)	<0.001	0.012 (0.007, 0.017)	<0.001
	P for trend		<0.001		<0.001		<0.001
lnTSH	Continuous	0.061 (0.029, 0.093)	<0.001	0.069 (0.035, 0.104)	<0.001	0.074 (0.034, 0.114)	<0.001
	Q4 vs. Q1	0.108 (0.049, 0.167)	<0.001	0.120 (0.057, 0.183)	<0.001	0.120 (0.047, 0.192)	0.001
	P for trend		<0.001		<0.001		0.002

CI, confidence interval; TyG, triglyceride-glucose index; FT3, free triiodothyronine; FT4, free thyroxine; TSH, thyroid-stimulating hormone; Data are presented as *β* coefficients (95% CI). Model 1: Crude model. Model 2: Adjusted for age and sex. Model 3: Adjusted for Model 2 plus BMI, smoking, drinking, education, ALT, AST, GGT, UA, Cr, BUN, Hb, TgAb, and TPOAb.

The TyG index was also positively associated with the FT3/FT4 ratio in the fully adjusted model (*β* = 0.004, 95% CI: 0.001, 0.007, *p* = 0.005). Participants in the highest TyG quartile had a higher FT3/FT4 ratio than those in the lowest quartile (*β* = 0.012, 95% CI: 0.007, 0.017, *p* < 0.001), with a significant trend across quartiles (P for trend < 0.001).

In addition, the TyG index was significantly and positively associated with lnTSH levels in the fully adjusted model (*β* = 0.074, 95% CI: 0.034, 0.114, *p* < 0.001). Participants in the Q4 group had higher lnTSH levels than those in the reference group (*β* = 0.120, 95% CI: 0.047, 0.192, *p* = 0.001), and a significant trend across quartiles was also observed (P for trend = 0.002).

### 3.3 Non-linear association and threshold effect analysis between the TyG index and thyroid function parameters

**Figure 1:**
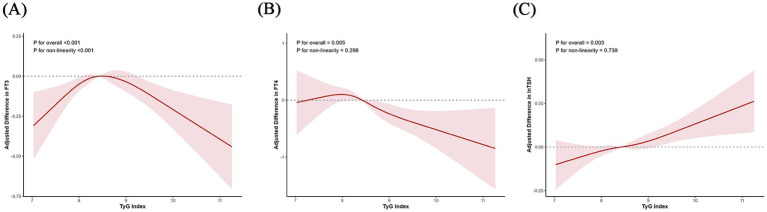
Restricted cubic spline analyses of the associations between the TyG index and (A) FT3, (B) FT4, and (C) lnTSH. The solid lines represent the multivariable-adjusted differences in predicted thyroid function parameters relative to the reference TyG value, and the shaded areas indicate 95% confidence intervals (95% CIs). The median TyG index was used as the reference value. P for overall association evaluates the overall significance of the association, and P for non-linearity assesses departure from a linear relationship. All models were fully adjusted for age, sex, BMI, smoking status, drinking status, education level, ALT, AST, GGT, UA, Cr, BUN, Hb, TgAb, and TPOAb.

To further explore potential non-linear associations between the TyG index and thyroid function parameters, RCS and threshold analyses were performed (Figure 1; Table 3). For FT3, the RCS analysis indicated a significant non-linear association between the TyG index and FT3 (P for overall < 0.001; P for non-linearity < 0.001). Piecewise linear regression identified an inflection point at 8.311. Below this value, the TyG index was positively associated with FT3 (*β* = 0.245, 95% CI: 0.107, 0.383, *p* < 0.001), whereas above this value, the association was negative (*β* = −0.122, 95% CI: −0.196, −0.048, *p* = 0.001), consistent with an inverted U-shaped pattern (P for likelihood ratio test <0.001).

**Table 3:** Threshold effects of the TyG index on FT3 and FT4 identified by multivariate piecewise linear regression.

Thyroid parameters	TyG index range	Adjusted *β* (95% CI)	*P*-value	LRT test
FT3				<0.001
	≤8.311	0.245 (0.107, 0.383)	<0.001	
	>8.311	−0.122 (−0.196, −0.048)	0.001	
FT4				0.016
	≤7.591	1.550 (0.042, 3.058)	0.044	
	>7.591	−0.340 (−0.504, −0.177)	<0.001	

Threshold effects of the TyG index on FT3 and FT4 were assessed using multivariable piecewise linear regression. Adjusted *β* coefficients with 95% CIs and p-values are presented for each TyG range. Models were adjusted for potential confounders. LRT was used to compare the piecewise linear model with the single linear model.

For FT4, although no statistically significant departure from linearity was detected in the RCS analysis (P for non-linearity = 0.298), the LRT suggested that the piecewise linear model provided a better fit than the single linear model (*p* = 0.016). The estimated inflection point was 7.591. The TyG index was positively associated with FT4 below 7.591 (*β* = 1.550, 95% CI: 0.042, 3.058, *p* = 0.044), whereas a significant negative association was observed above this value (*β* = −0.340, 95% CI: −0.504, −0.177, *p* < 0.001).

For lnTSH, the RCS analysis did not show evidence of a non-linear association with the TyG index (P for non-linearity = 0.895), which was consistent with the linear association observed in the multivariable linear regression analysis.

### 3.4 Subgroup analyses stratified by potential effect modifiers and sensitivity analyses

**Figure 2:**
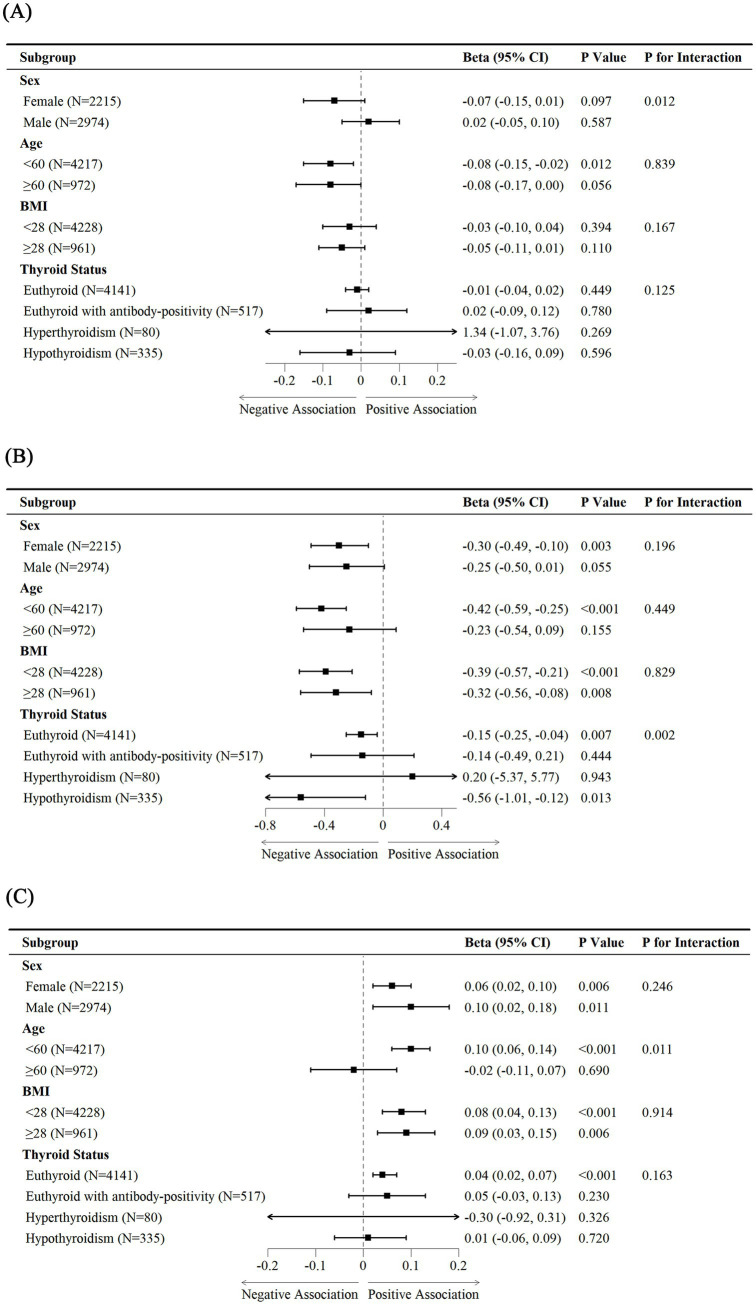
Subgroup analyses of the associations between the TyG index and thyroid function parameters. **(A)** Subgroup analysis of the association between the TyG index and FT3. **(B)** Subgroup analysis of the association between the TyG index and FT4. **(C)** Subgroup analysis of the association between the TyG index and TSH. The vertical dashed line represents the null value (*β* = 0). Subgroup analyses were performed according to sex, age, body mass index (BMI), and thyroid status. All models were adjusted for relevant confounding variables. *p* values indicate the significance of the association within each subgroup, whereas P for interaction indicates whether the association differed significantly across subgroups.

To assess the consistency of the associations between the TyG index and thyroid function parameters, analyses were further stratified by age, sex, BMI, and thyroid function status (Figure 2). For FT3, the association between the TyG index and FT3 differed by sex (P for interaction = 0.012). For FT4, thyroid function status significantly modified the association with the TyG index (P for interaction = 0.002). The inverse association was observed among euthyroid participants and appeared stronger among participants with hypothyroidism, whereas no clear association was observed in the hyperthyroid subgroup. For lnTSH, the interaction by age was statistically significant (P for interaction = 0.011), with a positive association observed among participants aged < 60 years but not among those aged ≥ 60 years. Overall, the inverse association with FT4 and the positive association with lnTSH were generally consistent across most strata, although significant interactions were observed in subgroup analyses.

Furthermore, sensitivity analyses were conducted in strictly euthyroid participants (excluding participants with thyroid diseases or medication use) to further assess the consistency of the observed associations (Supplementary Tables S2, S3). For FT4, the estimated inflection point remained similar to that observed in the primary analysis (7.560 in the euthyroid population versus 7.591 in the total population). The piecewise linear pattern, characterized by a positive association below the inflection point and a negative association above it, was supported by the LRT (*p* = 0.012). For FT3, although the estimated inflection point shifted to a higher TyG index in the strictly euthyroid participants (9.410 versus 8.311 in the primary analysis), the overall pattern remained consistent with an inverted U-shaped association. Specifically, a positive association was observed below the inflection point, whereas a negative association was observed above it (P for LRT < 0.001).

## 4 Discussion

This cross-sectional study examined the association between the TyG index, a surrogate marker of insulin resistance, and key thyroid function parameters. Our findings indicated that the TyG index was linearly and positively associated with TSH, whereas its associations with FT3 and FT4 exhibited non-linear patterns. Fully adjusted regression showed no statistically significant linear association between the TyG index and FT3, but RCS analyses suggested an inverted U-shaped association pattern with an inflection point at 8.311; therefore, a simple linear model may not fully characterize this association. Similarly, for FT4, while the global test for non-linearity did not reach statistical significance, piecewise linear regression suggested an inflection point at 7.591 with divergent slopes on either side (positive association below, negative association above), suggesting that localized differences in this association pattern may not be fully captured by global spline tests. Furthermore, possible effect modification was observed across key subgroups, including sex for FT3, thyroid function status for FT4, and age for TSH. Collectively, these results suggest a complex association between the TyG index and thyroid function parameters and underscore the importance of applying flexible, non-linear analytical approaches. Sensitivity analyses showed generally consistent associations after excluding participants with thyroid dysfunction and positive thyroid antibodies, suggesting that the observed metabolic-thyroid association was not confined to autoimmune pathology. Taken together, these findings describe cross-sectional association patterns between the TyG index and thyroid function parameters that require confirmation in longitudinal studies.

This study showed a positive linear association between the TyG index and TSH. This observation is in line with earlier population-based investigations reporting a positive association between TSH and insulin resistance-related indices, including the U.S. population study by Safari et al. (18), the study by Zhang et al. (19), and the Brazilian Healthy Adult Cohort Study (20). Regarding the potential biological underpinnings of this association, experimental studies have suggested that TSH may be linked to metabolic inflammation through TSH receptors expressed on macrophages (21). Specifically, endoplasmic reticulum stress associated with elevated TSH may be related to insulin resistance in adipose tissue. This process may also be accompanied by polarization of adipose tissue macrophages toward a pro-inflammatory M1 phenotype, with increased cytokine release (21, 22). Furthermore, TSH has been reported to be involved in lipolysis and insulin signaling regulation through protein kinase B phosphorylation (23). Although these experimental findings primarily illustrate potential links between TSH signaling and insulin resistance, they provide biological plausibility for the positive association observed between the TyG index and TSH in our cross-sectional analysis. Nevertheless, this cross-sectional analysis cannot determine whether metabolic abnormalities precede changes in TSH, whether thyroid-related alterations contribute to metabolic dysfunction, or whether both are influenced by shared confounding factors. Therefore, the proposed mechanisms should be interpreted cautiously.

Although a generally consistent positive linear association has been observed between the TyG index and TSH, findings regarding its associations with thyroid hormones, including FT3 and FT4, remain inconsistent across studies. Some studies have suggested that serum FT3 levels may be elevated in states of insulin resistance or metabolic abnormalities. Specifically, a positive association between FT3 and HOMA-IR was reported in a Sudanese cohort (24), while elevated FT3/FT4 ratios have been associated with visceral adiposity and insulin resistance in several studies (25–27). In the present study, we also examined the FT3/FT4 ratio to better characterize the relative balance between FT3 and FT4. The positive association between the TyG index and the FT3/FT4 ratio after full adjustment suggests that the metabolic-thyroid association may not be fully reflected by FT3 or FT4 alone. In contrast, several studies have reported an inverse association between serum FT4 and insulin resistance-related indices. This inverse association has been observed across diverse populations, including the Tehran Thyroid Study (28), the study by Choi et al. (29), the analysis conducted by Cheng et al. (30), and hospital-based populations (31). Notably, a small number of studies have also reported inverse associations of the TyG index with both FT3 and FT4 (32). Collectively, this heterogeneity suggests that simple linear models may be insufficient to fully reflect these associations. These associations may be influenced by multiple factors, underscoring the need to investigate non-linear patterns or potential threshold effects.

By employing RCS analysis and piecewise linear regression, we characterized the potential non-linear associations between thyroid hormones (FT3 and FT4) and the TyG index, identifying inflection points at 8.311 for FT3 and 7.591 for FT4. Interestingly, these estimated inflection points fall within a range similar to TyG index values reported in some studies of metabolic disorders. For instance, TyG index values have been reported in relation to cardiovascular events (8.84), all-cause mortality (9.05) (33), MASLD (9.26) (34), gallstone disease, and chronic kidney disease (8.98) (35, 36). This numerical similarity may provide contextual information for interpreting the observed TyG range, but this interpretation should be made cautiously. In the present study, these inflection points mainly describe the shape of the associations between the TyG index and thyroid function parameters and should not be regarded as validated clinical cut-offs. The observed inverted U-shaped association may indicate that the association between the TyG index and FT3 differs across TyG ranges, rather than providing evidence of a confirmed compensatory or decompensatory mechanism.

Several potential mechanisms may help explain the observed non-linear associations, although these explanations remain speculative. The thyroid gland primarily secretes thyroxine (T4), which is subsequently converted to the more metabolically active triiodothyronine (T3) in peripheral tissues via deiodinases (37, 38). At lower TyG index levels, the observed positive association with FT3 may reflect variation in peripheral thyroid hormone conversion. Specifically, some evidence suggests that in metabolically healthier states or conditions of mild insulin resistance, enhanced peripheral T4 to T3 conversion may represent a potential adaptive metabolic response related to metabolic homeostasis (39, 40). Conversely, at higher TyG index levels, the inverse associations may be related to the interplay of multiple factors. For instance, elevated TyG index, may be associated with alterations in the regulation of the hypothalamic–pituitary-thyroid (HPT) axis (41), a pro-inflammatory milieu (42), and cellular metabolic stress (43), all of which may be related to changes in thyroid hormone synthesis, conversion, and action, and may be compatible with lower FT3 and FT4 levels at higher TyG index levels. The precise underlying mechanisms require further clarification through experimental and longitudinal studies.

Although the overall association pattern between the TyG index and thyroid function parameters was relatively consistent, subgroup analyses suggested possible effect modification in specific populations. In individuals with normal thyroid function, the TyG index was significantly and inversely associated with FT4, and this inverse association appeared to be more pronounced in those with hypothyroidism. Previous studies have reported associations between the TyG index and thyroid function parameters, including a negative association with FT4 (44). This association appeared stronger in subclinical hypothyroidism (45). A Korean population-based cohort also reported that hypothyroidism was associated with elevated insulin resistance-related indicators (46). Regarding sex differences, large population-based studies have shown that women are more prone to thyroid dysfunction, with a stronger association between FT3 and metabolic syndrome risk (47). This suggests that sex may modify the association pattern between the TyG index and FT3 in relation to differences in thyroid hormone regulation and metabolic phenotype. The positive association between the TyG index and TSH was mainly observed in individuals aged <60 years and weakened in those aged ≥60 years. Spira et al. (48) reported that the association between thyroid function and insulin resistance was stronger in younger and middle-aged individuals but not in older populations, suggesting age as a potential modifier. Age-related alterations in thyroid hormone responsiveness and feedback control may contribute to differences in the metabolic-thyroid association. Overall, these findings suggest the complexity of metabolic-thyroid associations.

The major strengths of this study include the large sample size and the use of RCS together with piecewise linear regression to explore possible non-linear associations between the TyG index and thyroid function parameters. The consistency of our findings was supported by sensitivity analyses excluding participants with thyroid dysfunction and positive thyroid antibodies, suggesting that the observed metabolic-thyroid association was not solely driven by overt thyroid dysfunction or thyroid antibody positivity.

However, several limitations should be acknowledged. First, the cross-sectional design precludes causal or temporal inference, and therefore the temporal sequence between TyG index levels and differences in thyroid function parameters cannot be determined. Second, the findings from this Chinese population may not be readily generalizable to other ethnic groups. Third, several potentially relevant variables were unavailable or incompletely characterized, including dietary patterns, physical activity, menopausal status, iodine intake, socioeconomic factors, medication use beyond thyroid-related drugs, and long-term glycemic status. Although we adjusted for multiple confounders, residual confounding cannot be completely ruled out. In particular, HbA1c data were available only for a subset of participants, which limited our ability to distinguish normoglycemia, prediabetes, and diabetes across the entire study population. In the sensitivity analysis (Supplementary Table S4), further adjustment for HbA1c did not materially change the associations of the TyG index with FT4 and lnTSH, whereas the association with FT3 was attenuated and became non-significant. Future studies with complete HbA1c data and longitudinal glycemic assessments are warranted. Finally, although several associations reached statistical significance, the estimated differences in thyroid function parameters were relatively modest, and the identified inflection points were exploratory. Therefore, the clinical significance of the identified inflection points should be further assessed in independent prospective cohorts before they can be considered for clinical screening or risk stratification.

## 5 Conclusion

This study observed a complex association pattern between the TyG index and thyroid function, characterized by a linear positive association with TSH, an inverted U-shaped association with FT3, and a threshold-dependent association with FT4. These findings were generally supported by sensitivity analyses conducted among strictly euthyroid participants, with the FT3 pattern remaining consistent and the FT4 association remaining similar to that observed in the primary analysis. Furthermore, subgroup analyses suggested potential effect modification across several subgroups, including age, sex, and thyroid function status, indicating that the association between the TyG index and thyroid function may differ across specific populations. As a surrogate marker of insulin resistance, the TyG index may help characterize the association between metabolic status and thyroid function in the general population, but the clinical relevance of these findings requires further validation.

## Data Availability

The datasets presented in this article are not readily available because they are subject to privacy and ethical restrictions. Requests to access the datasets should be directed to XG, guanxiao@email.sdu.edu.cn.
