# Supplementary material for: Non-linear associations of the triglyceride-glucose index with thyroid function in a large Chinese population: a cross-sectional study
Source: Front Nutr. 2026 Jul 23;13:1841938. doi: 10.3389/fnut.2026.1841938 (PMC13442547; doi:10.3389/fnut.2026.1841938)
Supplement: Supplementary file 1 [file Table_1.DOCX]

**Supplementary Table 1. Additional baseline characteristics of the study participants stratified by TyG index quartiles.**

| Characteristics | TyG index | | | | Overall  (N=5189) | P-value |
| --- | --- | --- | --- | --- | --- | --- |
|  | Q1 (N=1298) | Q2 (N=1297) | Q3 (N=1297) | Q4 (N=1297) |  |  |
| Smoking Status |  |  |  |  |  | <0.001 |
| No | 1175 (90.5%) | 1121 (86.4%) | 1046 (80.6%) | 934 (72.0%) | 4276 (82.4%) |  |
| Yes | 93 (7.2%) | 149 (11.5%) | 220 (17.0%) | 330 (25.4%) | 792 (15.3%) |  |
| Drinking Status |  |  |  |  |  | <0.001 |
| No | 1057 (81.4%) | 961 (74.1%) | 848 (65.4%) | 711 (54.8%) | 3577 (68.9%) |  |
| Yes | 211 (16.3%) | 309 (23.8%) | 418 (32.2%) | 552 (42.6%) | 1490 (28.7%) |  |
| Education Level |  |  |  |  |  | 0.006 |
| ≤ High school | 102 (7.9%) | 131 (10.1%) | 131 (10.1%) | 154 (11.9%) | 518 (10.0%) |  |
| > High school | 1166 (89.8%) | 1136 (87.6%) | 1134 (87.4%) | 1101 (84.9%) | 4537 (87.4%) |  |
| Hb (g/L) | 137.8 ± 16.4 | 143.7 ± 16.9 | 149.8 ± 14.7 | 154.5 ± 13.2 | 146.4 ± 16.6 | <0.001 |
| ALT (U/L) | 12(9, 18) | 15(11, 22) | 19(14, 28) | 24(17, 38) | 17(12, 26) | <0.001 |
| AST (U/L) | 16(14, 20) | 18(15, 22) | 19(16, 23) | 21(17, 26) | 18(15, 23) | <0.001 |
| GGT (U/L) | 14(11, 19) | 19(14, 26) | 24(17, 35) | 36(24, 56) | 21(15, 34) | <0.001 |
| Cr (μmol/L) | 67.6 ± 13.2 | 71.5 ± 14.3 | 74.9 ± 14.3 | 76.3 ± 13.1 | 72.6 ± 14.2 | <0.001 |
| BUN (mmol/L) | 4.9 ± 1.2 | 5.1 ± 1.2 | 5.2 ± 1.2 | 5.3 ± 1.2 | 5.1 ± 1.2 | <0.001 |
| UA (μmol/L) | 287.0 ± 74.9 | 320.0 ± 80.9 | 347.7 ± 83.2 | 377.4 ± 83.2 | 333.0 ± 87.3 | <0.001 |
| FBG (mmol/L) | 4.4 ± 0.4 | 4.7 ± 0.6 | 4.9 ± 0.9 | 5.7 ± 1.6 | 4.9 ± 1.1 | <0.001 |
| TG (mmol/L) | 0.67(0.57, 0.78) | 1.03(0.93, 1.14) | 1.47(1.30, 1.66) | 2.43(1.99, 3.14) | 1.20(0.85, 1.79) | <0.001 |
| TC (mmol/L) | 4.5 ± 0.8 | 4.8 ± 0.9 | 5.0 ± 0.9 | 5.3 ± 1.0 | 4.9 ± 1.0 | <0.001 |
| HDL-C (mmol/L) | 1.5 ± 0.3 | 1.4 ± 0.3 | 1.3 ± 0.3 | 1.1 ± 0.3 | 1.3 ± 0.3 | <0.001 |
| LDL-C (mmol/L) | 2.6 ± 0.7 | 2.9 ± 0.7 | 3.2 ± 0.8 | 3.2 ± 0.8 | 3.0 ± 0.8 | <0.001 |

Continuous variables are presented as mean ± SD or median(interquartile range), and categorical variables are presented as number (percentage).Abbreviations: Hb, hemoglobin; ALT, alanine aminotransferase; AST, aspartate aminotransferase; GGT, gamma-glutamyl transferase; Cr, serum creatinine; BUN, blood urea nitrogen; UA, uric acid; FBG, fasting blood glucose; TG, triglycerides; TC, total cholesterol; HDL-C, high-density lipoprotein cholesterol; LDL-C, low-density lipoprotein cholesterol.

**Supplementary Table S2: Sensitivity Analysis of the Association Between TyG Index and Thyroid Function Parameters in Strictly Euthyroid Participants**

| Outcome | β | 95% CI | P Value |
| --- | --- | --- | --- |
| **FT3** | -0.012 | (-0.045, 0.021) | 0.472 |
| **FT4** | -0.140 | (-0.250, -0.030) | 0.012 |
| **lnTSH** | 0.043 | (0.016, 0.069) | 0.002 |

Euthyroid participants (N = 4141). Models were adjusted for Sex, Smoking, Drinking, Education, Age, BMI, ALT, AST, GGT, UA, CR, BUN, Hb, TgAb, and TPOAb.

**Supplementary Table S3**. Threshold effects of the TyG index on thyroid parameters in the euthyroid population (Sensitivity Analysis)****

| Thyroid  Parameters | TyG Index Range | Adjusted β (95% CI) | P-value | LRT test (P-value) |
| --- | --- | --- | --- | --- |
| **FT3** |  |  |  | **< 0.001** |
|  | ≤ 9.410 | 0.062 (0.021, 0.103) | 0.003 |  |
|  | > 9.410 | -0.263 (-0.354, -0.171) | < 0.001 |  |
| **FT4** |  |  |  | **0.012** |
|  | ≤ 7.560 | 1.267 (0.162, 2.372) | 0.025 |  |
|  | > 7.560 | -0.177 (-0.290, -0.063) | 0.002 |  |

Models were adjusted for age, sex, BMI, smoking, drinking, education level, ALT, AST, GGT, UA, Cr, BUN, Hb, TgAb, and TPOAb. The analysis was restricted to euthyroid participants(N = 4141). CI, confidence interval; TyG, triglyceride-glucose; LRT, likelihood ratio test.

**Supplementary Table S4. Sensitivity analysis of the associations between TyG index and thyroid function parameters in participants with available HbA1c data.**

| Outcome | Sensitivity Model 1  β (95% CI) | P value | Sensitivity Model 2  β (95% CI) | P value |
| --- | --- | --- | --- | --- |
| **FT3** | -0.086 (-0.166, -0.007) | 0.034 | -0.032 (-0.120, 0.057) | 0.482 |
| **FT4** | -0.267 (-0.499, -0.036) | 0.024 | -0.396 (-0.653, -0.139) | 0.003 |
| **lnTSH** | 0.091 (0.037, 0.145) | <0.001 | 0.080 (0.020, 0.140) | 0.009 |

Analyses were restricted to participants with available HbA1c data (N = 2499). TyG index was modeled as a continuous variable. Sensitivity Model 1 was adjusted for age, sex, BMI, smoking, drinking, education, ALT, AST, GGT, UA, Cr, BUN, Hb, TgAb, and TPOAb. Sensitivity Model 2 was additionally adjusted for HbA1c.
